# Violence-related measures against ambulance personnel: a study protocol for a scoping review with multi-framework conceptual mapping

**DOI:** 10.12688/hrbopenres.14505.1

**Published:** 2026-07-28

**Authors:** Samuel Fournier, Liz Kingston, Anna V. Chatzi

**Affiliations:** 1 University of Limerick Faculty of Education and Health Sciences, Limerick, County Limerick, Ireland; 2 École Supérieure d’Ambulancier et Soins d’Urgence Romande, Lausanne, Switzerland; 3 Health Research Institute, University of Limerick, Limerick, Ireland; 4 Centre for Implementation Research (CIRcUL), University of Limerick, Limerick, County Limerick, Ireland

**Keywords:** workplace violence, ambulance personnel, paramedics, emergency medical services, safety, human factors, ergonomics, scoping review

## Abstract

**Background:**

Violence against ambulance personnel is a recognised occupational hazard. Between 57% and 93% of prehospital responders report at least one episode of verbal or physical violence during their career. Despite this exposure, violence-related measures remain insufficiently mapped in the literature, and no review known to the authors has described their distribution across complementary safety science and human factors frameworks.

**Objectives:**

This scoping review aims to identify and map violence-related measures targeting ambulance personnel as reported in peer-reviewed literature, and to describe their distribution across five conceptual frameworks: levels of prevention, hierarchy of controls, Safety-I/Safety-II, typology of situations, and levels of the sociotechnical system. Sub-objectives address the isolated versus bundled nature of measures, their relation to the mission cycle, the types of violence targeted, and the proportion of measures that are insufficiently described for coding.

**Methods:**

The review follows JBI methodology for scoping reviews and will be reported according to PRISMA-ScR guidelines, which is adaption of the PRISMA extension for Scoping Reviews checklist. Eligibility criteria follow a Population – Concept – Context structure. The population is ambulance personnel involved in mission-related work. The concept covers violence-related measures targeting Type II and Type V violence. The context is prehospital ambulance services, including mission-related dispatch interfaces. Searches were conducted in PubMed/MEDLINE, Embase, CINAHL, Web of Science Core Collection and Scopus, covering English-language peer-reviewed sources published between 1 January 2014 and 15 May 2026. Title/abstract screening will be conducted by the principal investigator with calibrated eligibility criteria. Data will be extracted using a piloted charting form and coded according to a predefined codebook. The protocol is registered on the Open Science Framework ( 10.17605/OSF.IO/DYUWC).

## Introduction

Violence against healthcare workers is a well-documented occupational hazard, with consequences including physical injury, psychological distress, reduced job satisfaction, absenteeism and staff turnover ( International Labour Office ILO 2002; Lanctôt and Guay 2014). Meta-analyses estimate that the annual prevalence of workplace violence against healthcare professionals ranges from approximately 42% to 59%, with verbal abuse being the most commonly reported form and physical violence affecting approximately one in five workers ( Liu *et al.* 2019; Sahebi *et al.* 2022). These estimates are likely conservative because of persistent under-reporting and heterogeneous definitions across studies.

Ambulance personnel face specific exposure conditions. They work in private homes, public spaces, roadsides, and other uncontrolled environments, often in the presence of relatives or bystanders and under significant time pressure. Systematic reviews report that between 57% and 93% of prehospital responders have experienced at least one episode of verbal or physical violence during their career ( Murray *et al.* 2020). A meta-analysis specific to paramedics estimates a pooled prevalence of 66% for workplace violence, including 25% for physical violence, 58% for verbal abuse and 16% for sexual harassment ( Shabanikiya *et al.* 2021).

Despite this exposure, violence-related measures in the ambulance sector remain insufficiently mapped. Existing reviews have described the frequency, forms and consequences of violence, but they also highlight major gaps, including limited trend data, scarce economic impact, and a near absence of rigorous evaluations of prevention programmes ( Maguire *et al.* 2018; Murray *et al.* 2020). Hokee et al. (2023) have published a scoping review protocol on workplace violence towards paramedic personnel, signalling an emerging area of inquiry. However, no existing review appears to map violence-related measures across several safety sciences and ergonomics/human factors frameworks.

This scoping review aims to address this gap. It will identify violence-related measures reported in peer-reviewed literature and map them using five complementary frameworks: levels of prevention, hierarchy of controls, Safety-I/Safety-II, typology of situations and levels of the sociotechnical system. These frameworks are not used to evaluate effectiveness. They are used to describe what function a measure is intended to serve, what type of control it represents, what safety logic it reflects, what kind of threat situation it addresses, and which level(s) of the sociotechnical system are explicitly necessary for the measure to function. Their combination is intended to reveal blind spots that may remain invisible when using a single conceptual lens, such as an over-reliance on training or individual-level adaptation with limited upstream organisational, regulatory or inter-organisational controls.

## Protocol

### Protocol registration and version

This protocol and extended data (Codebook, search equations, PRISMA-ScR, and list of articles to test sensitivity) are available on the Open Science Framework: 10.17605/OSF.IO/DYUWC.

Any substantive amendments made after registration will be documented in the OSF record and reported in the final scoping review.

### Study type and methodological framework

This is a protocol for a scoping review with a protocol finalised before screening, full-text assessment, data extraction and synthesis. The methodological framework follows the JBI Manual for Evidence Synthesis, Chapter 10: Scoping Reviews, including the guidance on protocol development, eligibility criteria based on Population – Concept – Context (PCC), documented search strategy, source selection, data extraction and a descriptive synthesis ( Peters *et al.* 2024). The final review will be reported according to PRISMA-ScR ( Tricco *et al.* 2018).

The choice of a scoping review is justified by the expected diversity of document types, contexts and reported measures, as well as by the objective of mapping a field rather than comparatively evaluating the effectiveness of homogeneous interventions.

No formal critical appraisal of included sources will be conducted, as the objective of this scoping review is to map the available literature and conceptual distribution of measures rather than to assess intervention effectiveness.

### Operational definitions

**Violence.** Violence is defined as any incident occurring during ambulance work, including operational activities, mission-related dispatch and field response, involving physical aggression, threatening behaviour or verbal abuse, explicitly including incivility. Attacks on operational resources, such as sabotage or damage to an ambulance or equipment, are included where they form part of a pattern of threats or aggression towards the ambulance personnel, the service or what they represent. This definition aligns with the NIOSH framework for workplace violence ( National Institute for Occupational Safety and Health 2022).

**Scope of perpetrators.** The review is limited to Type II violence, committed by service users such as patients or relatives, and Type V violence, ideological or terrorist acts targeting responders, the organisation or its resources in the prehospital setting ( Howard 1996; American Nurses Association 2024). Type I, III and IV are excluded because they follow different causal dynamics and prevention logic from the external operational interface between ambulance personnel and service users or third parties along the mission cycle.

**Measure.** A measure is defined as a targeted action aimed at preventing, anticipating, detecting, managing or limiting the consequences of violence against ambulance personnel. A measure may stand alone or form an identifiable component of a bundle, programme or broader scheme. Measures may be designed, implemented or mobilised before, during or after exposure to violence, if they are explicitly related to ambulance work and the safety of ambulance personnel.

**Mission cycle and exposure pathway.** For analytical purposes, the mission cycle refers to the sequence of mission-related activity from the initial receipt of a call by dispatch through to the return to service after patient handover. The segmentation used in this review is adapted by the authors from the six-function structure of the Star of Life, the official symbol of emergency medical services designed and trademarked by the National Highway Traffic Safety Administration ( U.S. Department of Transportation 1995), which defines the core functions of EMS as detection, reporting, response, on-scene care, care in transit, and transfer to definitive care. Coding will use the following segments: pre-event, dispatch, approach/arrival, scene/patient care, transport, handover and post-event. The pre-event and post-event segments are not part of the institutional Star of Life structure but are added by the authors as exposure pathway segments to capture upstream prevention measures and post-incident recovery-oriented measures that fall outside the strict operational mission cycle.

### Aims and research questions

**Main objective.** To identify and map violence-related measures targeting ambulance personnel, as described in peer-reviewed scientific literature, and to describe their distribution according to five conceptual frameworks drawn from safety science and ergonomics/human factors.

**Main research question.** Which violence-related measures targeting ambulance personnel, including the mission-related dispatch interface, are described in English-language peer-reviewed literature published between 2014 and 2026, and how are these measures distributed across the levels of prevention, the hierarchy of controls, the Safety-I/Safety-II lens, the typology of situations and the level(s) of the sociotechnical system addressed?

Sub-questions are:

1. Are measures described primarily isolated actions or components of broader programmes or bundles?
2. To which segments of the mission cycle or exposure pathway do these measures relate?
3. What types of violence are specifically targeted by the measures?
4. For how many measures is the available description insufficient to complete the coding, and what does this gap suggest about the maturity and reporting quality of the evidence base?

### Conceptual orientation of the mapping

This review uses five complementary conceptual frameworks to map the extracted measures.

Table 1 summarises their role in the review. Detailed coding rules are available with the OSF registry of the protocol.

**Table 1.** Summary of the conceptual orientation of the mapping.

Framework	Coding focus	Role in the review
Levels of prevention	Primary, secondary or tertiary prevention.	Describes whether the measure acts before violence, during risk detection/escalation, or after the event.
Hierarchy of controls	Elimination, substitution, engineering/technical controls, administrative controls, personal protective equipment.	Describes the primary control mechanism and identifies dependence on lower-order controls such as training or equipment.
Safety-I/Safety-II	Predominantly Safety-I, predominantly Safety-II, mixed or indeterminable.	Describes whether the measure mainly controls failure and deviations or supports anticipation, adaptation and successful performance under variable conditions.
Typology of situations	Regular threat, irregular threat, unprecedented event or unspecified.	Describes the type of threat situation the measure is designed to address, where the source provides sufficient information.
Sociotechnical system level	Staff/work, management, company, regulators/associations, government.	Locates the system level(s) explicitly necessary for the measure to function. Inter-organisational interfaces will be recorded separately.

### Eligibility criteria

Eligibility criteria are structured according to the Population – Concept – Context framework. A source must describe at least one identifiable violence-related measure to be eligible.

The 2014 cut-off was selected to focus on contemporary literature aligned with the safety science and systems-oriented perspective informing the review. The year corresponds to the publication of Hollnagel’s Safety-I/Safety-II perspective, one of the conceptual frameworks used in this review ( Hollnagel 2014). A sensitivity check was conducted on three known relevant records published before 2014. None of these records appeared to contain violence-related measures unique to the pre-2014 literature. The list of records examined is provided through the OSF registration.

Table 2 summarises the eligibility criteria.

**Table 2.** Summary of the inclusion and exclusion criteria.

Domain	Inclusion	Main exclusions
Population	Ambulance personnel involved in mission-related work. Dispatch is treated as an operational interface when the measure explicitly concerns the safety of ambulance crews.	Professionals outside ambulance services without an explicit ambulance component; air ambulance.
Concept	Work-related violence against ambulance personnel, as operationally defined, and related measures to prevent, anticipate, detect, manage or mitigate its consequences.	Documents describing no eligible measure; violence outside the operational definition; violence outside the retained perpetrator scope.
Context	Prehospital ambulance services, including mission-related dispatch and relevant operational or inter-organisational interfaces explicitly linked to crew safety.	Hospital-only settings; non-ambulance settings; contexts not attributable to ambulance work.
Source types	Peer-reviewed empirical studies, reviews and peer-reviewed conference proceedings published in academic venues. Study protocols are eligible only if they describe at least one extractable violence-related measure.	Grey literature, reports, guidelines, theses, dissertations, press articles, editorials, commentaries, letters, corrigenda, errata and protocols without an extractable measure.
Language	English.	Other languages.
Period	1 January 2014 to 15 May 2026.	Outside the period.

### Information sources and search strategy

The following databases were searched: PubMed/MEDLINE, Embase, CINAHL, Web of Science Core Collection and Scopus. This combination was selected to cover biomedical, occupational health, paramedical, emergency medical services, safety sciences and multidisciplinary literature.

This strategy was developed in consultation with an academic librarian and structured around two clusters of terms:

• Ambulance, paramedic, emergency medical services and prehospital care.
• Workplace violence and violence.

No third cluster for intervention or measure terms was added. This decision is deliberate because terminology for violence-related measures is heterogeneous and because some relevant sources describe measures only outside the title or abstract. Adding a measure/intervention cluster at the search stage was judged likely to reduce sensitivity. Identification of sources describing eligible measures will therefore be carried out during screening and full-text assessment.

No peer-reviewed or publication-status filter will be applied in the database equations. Peer-review status and source type will be assessed during screening and eligibility assessment because filters are inconsistent across databases. The complete database-specific search strategies are available through the OSF registry.

The PubMed/MEDLINE strategy was:

(“Ambulances”[Mesh] OR “Paramedics”[Mesh] OR “Emergency Medical Technicians”[Mesh] OR “Emergency Medical Services”[Mesh] OR ambulance*[tiab] OR paramedic*[tiab] OR “emergency medical technician*”[tiab] OR “emergency medical service*”[tiab] OR prehospital [tiab] OR “pre-hospital”[tiab]) AND (“Workplace Violence”[Mesh] OR “Violence”[Mesh] OR violen*[tiab]) AND english [lang] AND (“2014/01/01”[Date - Publication]: “2026/05/15”[Date - Publication])

The PubMed strategy was iteratively tested against a validation set of 57 PubMed-indexed records known to be relevant to the review topic. The final PubMed strategy retrieved all validation records aligned with the review scope and publication period. Strategies for the other databases were adapted to their syntax and indexing structures and tested against the validation set. Where exact date limits were unavailable, year-based limits were used and records published after 15 May 2026 will be excluded during eligibility assessment. Any additional source identification method will be documented in the final PRISMA-ScR flow diagram.

### Source selection process

References will be imported and deduplicated into EndNote. Title/abstract records and full text will be then screened. The final review will report the software used, the deduplication procedure and the numbers of records at each stage in a PRISMA-ScR flow diagram.

Screening will be conducted in two phases. In the first phase, title and abstract screening will be conducted by the principal investigator (SF). Co-authors (AC, LK) will be consulted for uncertain cases; discrepancies will be resolved by consensus and documented in a methodological decision log. In the second phase, full-text screening will begin with an independent pilot conducted by SF and one co-author (AC) on an initial sample of 20 records. Screening decisions will be compared, eligibility criteria refined as needed, and any disagreements resolved by discussion before SF proceeds with single-reviewer full-text screening of the remaining records. Co-authors (AC, LK) will remain available for consultation on uncertain full-text cases throughout this phase. Reasons for exclusion at the full-text stage will be documented using standardised exclusion categories: wrong population, wrong context, no eligible violence-related measure, violence outside the retained perpetrator scope, non-peer-reviewed source, grey literature, editorial/commentary/letter, corrigendum/erratum only, protocol without extractable measure, non-English language and outside publication period. All screening decisions, borderline cases, rule refinements and any deviations from the registered protocol will be recorded in a methodological decision log.

### Data charting and extraction

The primary unit of extraction will be the measure, as defined above. Where a document describes a programme, bundle or broader scheme comprising several distinct components, each component will be extracted individually if it can be identified as a targeted action relevant to preventing, anticipating, detecting, managing or mitigating violence against ambulance personnel. The affiliation of each extracted measure to a broader bundle or programme will be retained.

For a source to be included, it must describe at least one identifiable violence-related measure. Sources that merely mention the need for prevention or management without describing any identifiable measure will be excluded. Within an included source, brief or insufficiently described measures may be retained in the extraction database and classified as non-codable where they cannot be reliably mapped across the conceptual dimensions.

Where a measure is described in both a primary source and a secondary source, extraction will be undertaken from the primary source whenever possible. If the primary source is not available in the corpus, extraction may be undertaken from the secondary source where the description is sufficiently detailed to support coding. When the same measure is described in more than one source, repeated descriptions will not be counted as independent measures unless they refer to distinct implementations, adaptations or contexts.

The extraction grid will be tested before full extraction and adjusted if necessary. A single master spreadsheet will be used for the descriptive extraction and conceptual coding. Extraction will distinguish between the stage at which a measure is designed, implemented or delivered and the point in the mission cycle or exposure pathway at which the measure is intended to act.

Table 3 summarises the key variables. Sex and gender will not be used as primary organising variables in this review because the unit of extraction is the violence-related measure rather than individual participants. Where included sources report sex- or gender-specific information that is directly relevant to the design, targeting, implementation or interpretation of a measure, this information will be charted when available.

**Table 3.** Structure of the master extraction table.

Block	Key variables
Source characteristics	Source ID; author; year; country; document type; study design; source type (primary/secondary).
Context and violence-related background	Ambulance setting/context; mission-related interface(s) involved; perpetrator scope addressed; type of violence targeted.
Measure description	Measure ID; short label; verbatim extract; isolated measure; bundle status; implementation or delivery timing; intended point of action; extraction source.
Coding status	Codable/non-codable; reason if non-codable; coding confidence; inference used.
Conceptual coding	Prevention level; hierarchy of controls; Safety-I/Safety-II; typology of situations; sociotechnical system level.
Notes	Analytical comments; ambiguities; coding decisions; rationale for borderline cases.

### Conceptual coding and reliability

The five conceptual dimensions are operationalised in the codebook (see OSF registry) that is defined before full extraction. Coding will be based primarily on explicit source text. Inference will be permitted only where specified in the codebook, and any inference will be documented in the coding comments.

An initial calibration exercise involving triple coding of selected articles will be carried out by SF and co-authors. Calibration will cover the five conceptual dimensions, as well as implementation timing, intended point of action, use of inference, and the distinction between codable and non-codable measures. Discrepancies will be resolved through consensus and, if necessary, the codebook will be revised and affected items recoded. After calibration, SF will conduct the remaining coding independently, with joint arbitration for doubtful cases. A decision log will document rule adjustments, borderline cases and interpretative decisions.

### Synthesis of results

The synthesis will follow a descriptive and cartographic approach. Included sources will first be summarised using descriptive frequencies, including distribution by year, country, document type and study design. Measures will then be summarised according to their descriptive characteristics: type of violence targeted, perpetrator scope, interfaces involved, isolated measures or bundle components, implemented/delivery timing, and intended point of action in the mission cycle or exposure pathway.

Extracted measures will then be charted across the five conceptual frameworks. The synthesis will combine numerical summaries with matrix-based mapping, including cross-tabulations where relevant, such as prevention level × hierarchy of controls, sociotechnical level × intended point of action, Safety-I/Safety-II × type of violence, and implementation timing × prevention level.

A narrative summary will accompany the tables and matrices. This summary will interpret concentrations, imbalances and blind spots in the literature, such as the over-representation of certain types of measures, the under-specification of their conditions of use, or the limited representation of upstream organisational or regulatory approaches. Measures that are extractable but not fully codable will be retained and reported separately. The proportion of non-codable measures and the reasons for non-codability will be treated as findings, reflecting the maturity and reporting quality of the evidence base.

### Ethics and dissemination

Ethical approval is not required for this scoping review because it uses publicly available published literature and does not involve human participants, identifiable personal data or animal research. The results will be disseminated through a peer-reviewed scoping review article and will inform subsequent work on the management of violence in ambulance services. The registered protocol, codebook and full search strategies are available on OSF as extended data. The extraction template, decision log template, reporting checklist and validation set for search sensitivity testing will be deposited on OSF when finalised. Findings may also be shared with scientific and professional audiences concerned with occupational safety, emergency medical services and human factors/ergonomics.

## Discussion

### Expected contribution

This scoping review will provide a structured empirical map of the violence-related measures reported in the literature and will describe their conceptual distribution. For practice, identifying systematic gaps such as over-reliance on training-based or administrative measures may support the design of more balanced and multi-level strategies. For research, the mapping will highlight where evidence is sparse, where descriptions of measures are insufficient for coding, and where future primary studies and intervention evaluations are needed.

The contribution of the review is not to determine which measures are effective, but to clarify what measures are described, how they are distributed across safety science and human factors frameworks, and which system levels and threat situations remain under-specified. This is intended to create a structured basis for subsequent empirical and applied research on violence management in ambulance services.

### Anticipated limitations

1. The literature may exhibit terminological heterogeneity, variable descriptive detail, and limited precision regarding the mechanisms, timing or intended function of measures. Some measures may therefore be extractable but not fully codable.
2. The restriction to publications from 2014 onward may exclude earlier studies. This is mitigated by a sensitivity check of known relevant pre-2014 records, which did not identify unique violence-related measures absent from later literature.
3. The restriction to English-language publications may exclude relevant literature in other languages, particularly French and German. This limits the capture of some European professional and institutional contexts.
4. Exclusion of grey literature, guidelines, professional reports and institutional policies may omit operational measures that exist in practice but have not been published in academic journals. This limitation is accepted to keep the review manageable and to maintain a consistent source-type threshold.
5. The review maps what is described in peer-reviewed literature, not what exists in ambulance services. Absence of a measure from the literature will not be interpreted as absence from practice.
6. Title/abstract screening and most of the full-text screening will be conducted by one reviewer (SF), which departs from JBI guidance recommending independent dual screening throughout. This is a significant methodological limitation. It is partially mitigated by: (a) co-author consultation for uncertain cases at both screening phases; (b) an independent pilot full-text screening conducted jointly by SF and one co-author on an initial sample, followed by discussion and refinement of eligibility criteria before single-reviewer screening resumes; and (c) a methodological decision log documenting all screening decisions and rule refinements. These measures reduce but do not eliminate the risk of selection bias introduced by single-reviewer screening.
7. The search strategy focuses on violence-related terminology and may miss sources that describe relevant measures using alternative terms without violence-related keywords. This risk is mitigated by multi-database searching and validation against known records.
8. Type V violence is included conceptually, but the search strategy was not designed as an exhaustive terrorism or targeted-attack search. Type V-related measures will be included where they are identified through the general violence search strategy or supplementary searching.

## AI declaration

Artificial intelligence (Microsoft Copilot) was used solely to improve the grammar, syntax, and readability of the manuscript. The authors take full responsibility for the content, interpretation, and conclusions presented.

## Data Availability

No data are associated with this article. Centre for Open Science (OSF). Title of dataset: Violence-related measures against ambulance personnel: a scoping review with multi-framework conceptual mapping https://doi.org/10.17605/OSF.IO/DYUWC ( Fournier, S., Kingston, L. and Chatzi, A. (2026)). This project contains the following underlying extended data:
• Codebook (Description: General coding principles). • Search equations (Description: Search and MESH terms for all Databases to be searched). • 260703 - PRISMA-ScR adapted protocol checklist completed (Description: Preferred reporting items for systematic reviews and meta-analysis extension for scoping reviews (PRISMA-ScR): adapted checklist for a scoping review protocol). • 260727 - List of the articles used to test the sensitivity of the equation.pdf. (Description: List of the articles used to test the sensitivity of the equation). Data is available under the terms of the CC0 1.0 Universal license. Protocol was developed in accordance with JBI guidance for scoping review protocols. The final scoping review will be reported according to PRISMA-ScR.
