# Supplementary material for: Violence-related measures against ambulance personnel: a study protocol for a scoping review with multi-framework conceptual mapping
Source: HRB Open Res. 2026 Jul 28;9:78. [Version 1] doi: 10.12688/hrbopenres.14505.1 (PMC13722857; doi:10.12688/hrbopenres.14505.1)
Supplement: Peer review history

# Reviewer report: Reviewer response for version 1

**DOI:** 10.21956/hrbopenres.15985.r57979

**Published:** 2026-10-30

**Authors:** Margaret Hodgins

**Affiliations:** 1 University of Galway, Galway, County Galway, Ireland

This scoping review protocol aims to identify and map violence-related measures targeting pre-hospital ambulance personnel, as reported in the peer reviewed literature. It adheres to the JBI methodology for scoping review. It is  presented in a clear and coherent manner ,  the protocol is robust and the review has the potential to make a significant contribution to this literature. Terms are clearly defined and the research questions are articulated well . The framework, search strategy and inclusion/exclusion criteria are described in adequate detail.

Is the study design appropriate for the research question?

Yes

Is the rationale for, and objectives of, the study clearly described?

Yes

Are sufficient details of the methods provided to allow replication by others?

Yes

Are the datasets clearly presented in a useable and accessible format?

Not applicable

Reviewer Expertise:

Workplace violence

I confirm that I have read this submission and believe that I have an appropriate level of expertise to confirm that it is of an acceptable scientific standard.

# Reviewer report: Reviewer response for version 1

**DOI:** 10.21956/hrbopenres.15985.r56925

**Published:** 2026-08-27

**Authors:** Brian J. Maguire

**Affiliations:** 1 Central Queensland University School of Health Medical and Applied Sciences, Rockhampton, Queensland, Australia

Study Protocol. Violence-related measures against ambulance personnel: a study protocol for a scoping review with multi-framework conceptual mapping [version 1; peer review: awaiting peer review]

 Summary

 The authors note that violence against ambulance personnel is a serious occupational hazard. Further, the specific risks for violence against these personnel are unique from other healthcare workers in that the ambulance personnel are interacting with patients and the public in times of extreme stress and in environments that are often uncontrollable. Studies have shown that large proportions of ambulance personnel describe having experienced violent encounters while on duty.

 The authors go on to note that although a number of studies have described their risks, there remains a paucity of information on violence-related measures for these professionals.

 Approach

 The authors will limit their reviews to peer-reviewed literature and use that to map five complementary frameworks. The main research question is: “Which violence-related measures targeting ambulance personnel, including the mission-related dispatch interface, are described in English-language peer-reviewed literature published between 2014 and 2026, and how are these measures distributed across the levels of prevention, the hierarchy of controls, the Safety-I/Safety-II lens, the typology of situations and the level(s) of the sociotechnical system addressed?”

 I think the eligibility criteria are mostly reasonable although it may be an unnecessary restriction to limit the search to no earlier than 2014. It is likely that the pool of eligible articles will be small to begin with, so including more years might improve the depth and breadth of the examined literature. In addition, the limitation may result in foundational approaches to violence prevention being omitted.

 The search strategy, selection process, data charting, coding and synthesis are all reasonable approaches. The process allows for explorations of various intervention type projects such as:  legal interventions, both aimed at increasing penalties for perpetrators and laws aimed at requiring agencies to provide specific training or equipment; engineering type interventions such as vests, and pepper spray; and educational type interventions such as training ambulance personnel in self-defense, or training the community in not attacking the ambulance personnel.

 I believe this project has the potential to make a meaningful contribution to occupational safety for EMS professionals.

 The reviewer used OpenAI’s ChatGPT (GPT-5.6 Sol) for editorial assistance including grammar and spelling. The reviewer independently evaluated all resulting content.

Is the study design appropriate for the research question?

Yes

Is the rationale for, and objectives of, the study clearly described?

Yes

Are sufficient details of the methods provided to allow replication by others?

Yes

Are the datasets clearly presented in a useable and accessible format?

Yes

Reviewer Expertise:

Epidemiology, occupational safety, emergency medical services

I confirm that I have read this submission and believe that I have an appropriate level of expertise to confirm that it is of an acceptable scientific standard.
